# Knowledge of healthcare providers regarding breastfeeding preterm infants in mainland China

**DOI:** 10.1186/s12887-018-1223-7

**Published:** 2018-07-31

**Authors:** Yuanyuan Yang, Rui Li, Jing Wang, Qingying Huang, Hong Lu

**Affiliations:** 1 Peking University School of Nursing, #38 Xueyuan Road, Haidian District, Beijing, 100191 China; 2 Peking University Third Hospital, Beijing, China

**Keywords:** Preterm infants; breastfeeding, Knowledge, Training needs, NICU, Healthcare providers

## Abstract

**Background:**

Support from healthcare professionals has been identified as an important variable affecting successful breastfeeding in preterm infants. This study aimed to explore the knowledge of healthcare providers regarding breastfeeding preterm infants in mainland China.

**Methods:**

An online cross-sectional survey of healthcare providers from 9 tertiary level neonatal intensive care units across 4 districts in mainland China was conducted from May–November, 2017. A total of 187 healthcare providers responded to the survey. Data included demographic information and current and desired knowledge about breastfeeding preterm infants. Breastfeeding knowledge was evaluated using a researcher-developed questionnaire with six domains based on breastfeeding preterm infant guidelines.

**Results:**

The average percent of correctly answered questions was 53.73%. The domain with the highest mean percent was ‘indications and methods of breastfeeding’ (64.41%). The lowest scoring domain was ‘characteristics of premature human milk’ (38.83%). Knowledge score was related to the following factors: age, profession, professional title, education background and offspring amount by univariate analysis (*p* < 0.05). Multiple linear regression analysis found that healthcare provider breastfeeding knowledge was associated with profession (physician or nurse), professional title, sex and total offspring. In terms of training needs, 86.7% of healthcare providers reported insufficient knowledge about breastfeeding in the preterm infant population; 82.2% wanted more knowledge about indications and contraindications related to breastfeeding premature infant; and 71.7% considered expert lectures as the most effective way of acquiring additional breastfeeding knowledge.

**Conclusion:**

The knowledge about breastfeeding in the preterm infant population among NICU healthcare professionals in mainland China was limited. More targeted training is needed to improve NICU healthcare provider knowledge regarding breastfeeding preterm infants.

## Background

According to the report released by the World Health Organization (WHO) in 2012, the incidence of preterm birth is 5 to 18% worldwide. More than 15 million premature infants are born every year, accounting for 11.1% of the total number of births worldwide [1]. In China, there are about 1.17 million premature infants born each year and the premature birth rate is approximately 7.1% [2]. Health benefits associated with breastfeeding preterm infants have been documented consistently; however, the success rates of breastfeeding preterm infants remains below desirable levels in China. More specifically a national investigation in 2009 of Chinese preterm infants reported less than 15% preterm infants received human milk while cared for in Neonatal Intensive Care Units (NICU) [3].

Support from healthcare professionals has been identified as an important variable affecting rates of successful breastfeeding in the preterm infant population [4]. Mothers view providers in the NICU as experts in infant care and look to them for guidance and support during hospitalization of their preterm infants. Thus, health care providers can have a significant impact on mother’s breastfeeding behaviors through education, support, and guidance. However, if NICU healthcare providers lack the necessary knowledge and skills about breastfeeding to provide mothers with support, then this may hinder successful breastfeeding in preterm infants [5–7].

If there is insufficient breastfeeding knowledge among health professionals, it is essential to provide breastfeeding education and training to improve knowledge, which will assist in the management of breastfeeding. Several studies explored the effectiveness of professional training to promote the onset or duration of breastfeeding. Results indicate that training healthcare professionals is an effective strategy in improving breastfeeding knowledge, skills, and practices and increases breastfeeding rates in preterm infants [8–10].

However, there is little evidence currently available related to the knowledge of healthcare providers regarding breastfeeding preterm infants in mainland China. Our study seeks to address this gap. With the findings, researchers and clinical experts can implement targeted training to enhance healthcare professionals’ breastfeeding knowledge, thereby increasing breastfeeding among mothers of preterm infants in China. Therefore, the purpose of this study was to assess current and needed knowledge about breastfeeding in the preterm infant population among NICU healthcare providers in mainland China.

## Methods

This study was approved by Peking University Institutional Review Board. An online questionnaire was sent to NICU healthcare providers from 9 tertiary care hospitals in 4 districts in mainland China by means of convenience sampling from May to November 2017. The informed consent was included with the survey and they were informed that their participation was anonymous and voluntary. If they agreed to answer the survey, the online format required them to answer all the questions. They were asked to answer the questions independently and to not access reference materials or ask others for help. The survey took approximately 10–15 min to complete.

### Instruments

The survey developed for this investigation was created by the research team based on results from literature reviews. The survey included three sections: (1) demographic information, (2) breastfeeding knowledge in the preterm infant population (knowledge questionnaire), (3) training assessment related to breastfeeding preterm infants. Demographics included age, sex, education, professional title, work experience in the NICU, professional role, institution, total offspring, and personal breastfeeding experience.

The second section, the knowledge questionnaire was a researcher developed survey to assess current knowledge about breastfeeding preterm infants. For this study, breastfeeding was defined as being human milk fed regardless of method. There were 37 ‘true/false/uncertain’ questions and 8 multiple-choice questions, for a total of 45 questions covering six domains. Correct answers were scored as 1 and incorrect or uncertain answers as 0. Respondents received a total score ranging from 0 to 45, with higher scores indicating more accurate knowledge of breastfeeding preterm infants.

The domains of the breastfeeding knowledge questionnaire was developed by the researchers after a review of current literature and guidelines about breastfeeding in the preterm infant population. Several sources were reviewed for content correctness and confirmation, such as CSPEN guidelines for nutrition support in neonates [11], recommendations for promoting breastfeeding in preterm infants in neonatal intensive care units (in Chinese) [12], and guidelines on optimal feeding of low birth weight infants in low-and middle-income countries [13]. Six experts in breastfeeding preterm infant were consulted to review the validity of the questionnaire. The content validity index (CVI) of the questionnaire was 0.965. Then, the questionnaire was piloted with 30 NICU healthcare providers to further identify the reliability. Cronbach’s alpha was used to measure the reliability. From this analysis, the Cronbach’s alpha of the breastfeeding knowledge questionnaire was 0.941. The Cronbach’s alpha for each domain can be found in Table 1.

**Table 1:** Domains and reliability of the knowledge questionnaire

Domains	Number of questions	Cronbach’s alpha
1. Indications and methods for breastfeeding preterm infants	17	0.719
2. Storage and transportation of human milk	6	0.862
3. Indications and contraindications for breastfeeding preterm infants	3	0.764
4. Use of human milk fortifier	8	0.852
5. Characteristics of digestive system and nutrient requirements of preterm infants	5	0.789
6. Characteristics of preterm human milk	6	0.884

### Statistical analysis

Data were presented as the mean ± standard deviation (SD) or n (percentage). Independent sample t-test and one-way analysis of variance (ANOVA) were performed to test differences among groups. Stepwise multiple linear regression model was used to analyze the associations between demographics and healthcare providers’ breastfeeding knowledge. Data was analyzed using the statistical software SPSS 19.0. Statistical significance was considered if *p* < 0.05.

## Results

### Demographic characteristics

A total of 187 healthcare providers responded to the survey. Data from 7 participants were excluded from data analysis because answers in each of the survey were the same, indicating a potential issue with data validity. Thus, the total sample for data analysis was 180.

The average age of participants was 30.48 ± 6.76 years old with an average work experience in the NICU of 5.33 ± 4.48 years. Additional demographic information of the NICU healthcare providers are shown in Table 2.

**Table 2:** Participant Demographics

Healthcare providers (*n* = 180)	*n*	%	Healthcare providers (*n* = 180)	*n*	%
Age (years)			Sex		
≤ 25	45	25.0	Male	6	3.3
26–30	66	36.7	Female	174	96.7
>30	69	38.3	Institution		
Professional title^a^			General hospital	145	80.6
Junior	144	80.0	Specialized hospital	35	19.4
Intermediate	31	17.2	Leaders		
Senior	5	2.8	Yes	14	7.8
Years of service in NICU			No	166	92.2
≤ 5	113	62.8	Total Offspring		
6–10	45	25.0	None	95	52.8
>10	22	12.2	One	76	42.2
Educational Background			Two or more	9	5.0
Junior college	62	34.4	Preterm offspring		
Undergraduate	99	55.0	Yes	5	2.8
Postgraduate or PhD	19	10.6	No	175	97.2
Profession			Personal breastfeeding experience		
Physician	35	19.4	Yes	80	44.4
Nurse	145	80.6	No	5	2.8

Note.^a^ Junior title refers to junior nurse and residency; intermediate refers to nurse-in-charge and attending physicians; Senior title refers to director physician, assistant director physician, director nurse and assistant director nurse

### Current knowledge about breastfeeding preterm infants

The average breastfeeding knowledge total score was 24.18 ± 4.17 with the range of scores from 34 to 12, highest to lowest respectively. The mean percentage of healthcare providers who correctly answered questions was 53.73%. The majority of the respondents (79.4%) had a score that was 60% (27/45) or below. Correctness and average score in six domains of the knowledge questionnaire were shown in order in Table 3.

**Table 3:** Knowledge regarding breastfeeding preterm infants in NICU Health Professionals

Domains	Number of items	Highest score	Lowest score	Score (Mean ± SD)	Accuracy (%)
1. Indications and methods for breastfeeding preterm infants	17	16	3	10.95 ± 2.20	64.41
2. Storage and transportation of human milk	6	6	1	3.15 ± 0.98	52.50
3. Indications and contraindications for breastfeeding preterm infants	3	3	0	1.55 ± 0.77	51.67
4. Use of human milk fortifier	8	8	1	3.93 ± 1.36	49.13
5. Characteristics of digestive system and nutrient requirements of preterm infants	5	5	0	2.26 ± 0.93	45.20
6. Characteristics of preterm human milk	6	3	0	2.33 ± 0.69	38.83

### Factors related to breastfeeding knowledge

Breastfeeding knowledge was significantly related to age, profession, professional title, educational background and total offspring through independent sample t-test and one-way ANOVA analysis (see Table 4).

**Table 4:** Related factors to breastfeeding knowledge of healthcare providers

Healthcare providers (*n* = 180)	Score (Mean ± SD)	t/F	*P*
Age (years)		9.606^△^	0.000
≤ 25	22.36 ± 3.84		
26–30	23.33 ± 3.61		
>30	25.65 ± 4.40		
Profession		−4.502^a^	0.000
Nurse	23.57 ± 3.09		
Physician	26.69 ± 3.56		
Professional title		8.710^a^	0.000
Junior	23.56 ± 3.91		
Intermediate	26.61 ± 4.38		
Senior	27.00 ± 4.36		
Educational Background		9.101^b^	0.000
Junior college	23.16 ± 4.12		
Undergraduate	24.15 ± 4.10		
Postgraduate or PhD	27.63 ± 2.81		
Total Offspring		6.748^b^	0.001
None	23.24 ± 3.45		
One	24.97 ± 4.78		
Two or more	27.33 ± 2.78		
Work Experience in the NICU		1.754^b^	0.176
≤ 5	23.93 ± 3.83		
6–10	24.04 ± 5.07		
>10	25.73 ± 3.67		
Personal breastfeeding experience		0.747^a^	0.493
Yes	25.33 ± 4.65		
No	23.60 ± 5.03		
Sex		−1.342^a^	0.236
Female	21.17 ± 5.64		
Male	24.28 ± 4.10		

Note. ^a^ refers to t value; ^b^ refers to F value

### Associations between breastfeeding knowledge score and demographic parameters

Stepwise multiple linear regression analysis was used to examine the associations between breastfeeding knowledge total score and demographics (i.e. age, sex, profession, professional title, educational background, institution, years of service in NICU, personal breastfeeding experience, total offspring, preterm offspring, and breastfeeding training experiences) of healthcare providers. Breastfeeding knowledge total score was the dependent variable and demographics were the independent variables. The equation that optimizes the model for the relationship with the highest multiple correlation coefficient (R^2^) was shown below:

$$ \mathrm{Breastfeeding}\ \mathrm{knowledge}\ \mathrm{score}=10.457+{0.248}^{\ast }\ \mathrm{Profession}+{0.141}^{\ast }\ \mathrm{Total}\ \mathrm{Offspring}+0.16{1}^{\ast}\mathrm{Sex}+{0.165}^{\ast }\ \mathrm{Profession}\mathrm{al}\ \mathrm{title} $$

$$ \mathrm{F}=9.598,p<0.01,\mathrm{adjusted}\ {\mathrm{R}}^{2=}0.161 $$

After adjustment of potential confounders, breastfeeding knowledge was significantly associated with profession, professional title and sex, whereas, not significantly associated with total offspring (*p* = 0.064). The R^2^ statistic represents these four independent variables in the equation explain 16.1% of the overall variation of the breastfeeding knowledge score. The results of the regressions were presented as constant, standard partial regression coefficient (β) and values (see Table 5).

**Table 5:** Associations between Breastfeeding Knowledge Score and Demographic Parameters

	β	SE^a^	t	*p*
Constant	10.457	3.437	3.042	0.003
Profession	0.248	0.762	3.428	0.001
Total Offspring	0.141	0.533	1.861	0.064
Sex	0.161	1.622	2.301	0.023
Professional title	0.165	0.670	2.132	0.034

^a^SE refers to standard error

### Training needs to gain breastfeeding knowledge for preterm infants

Both nurses and physicians reported a desire for more breastfeeding training (86.2% vs. 88.6%). More nurses than physicians reported having received breastfeeding training in the last year. (see Table 6).

**Table 6:** Training experience and needs on breastfeeding knowledge

	Desire more training	No training needed	*p*	Training experience	No training experience	*p*
	*n* (%)	*n* (%)		*n* (%)	*n* (%)	
Nurse	125 (86.2)	20 (13.8)	0.759	126 (86.9)	19 (13.1)	0.000
Physician	31 (88.6)	4 (11.4)		20 (57.1)	15 (42.9)	

### Developing knowledge about breastfeeding preterm infants

The top three ways to obtain knowledge about breastfeeding preterm infants were as follows: ward rounds (82.8%), expert lectures (66.7%) and literature queries (60.6%). Most healthcare providers (71.7%) reported expert lectures as the most effective way to access to breastfeeding knowledge, followed by academic communication (56.7%) and ward rounds (48.9%), as shown in Table 7.

**Table 7:** Access to knowledge on breastfeeding preterm infants

	Ways of access		Effective ways	
	*n*	%	*n*	%
Ward rounds	149	82.8	88	48.9
Expert lectures	120	66.7	129	71.7
Literature queries	109	60.6	69	38.3
Reference books	89	49.4	77	42.8
Online education	79	43.9	61	33.9
Academic communication	55	30.6	102	56.7
Visiting advanced units	38	21.1	51	28.3

### Training needed to develop knowledge about breastfeeding preterm infants

A desire for more knowledge about indications and contraindications for breastfeeding premature infants was most frequently reported by 82.2% of healthcare providers; while knowledge about methods to promote breastfeeding in preterm infants was not a topic that training was necessary (48.9%) (see Table 8).

**Table 8:** Knowledge requested for breastfeeding preterm infants

	Number	Percent
Indications and contraindications to breastfeeding preterm infants	148	82.2
Characteristics of digestive system and nutrient requirements of preterm infants	145	80.6
Breastfeeding methods for preterm infants	133	73.9
Nutrients, functions and mechanisms of premature breast milk	126	70.0
Methods of supplementing other nutrients during breastfeeding	125	69.4
Storage and transportation of breast milk	117	65.0
Methods to promote breastfeeding in preterm infants	88	48.9

## Discussion

The present study described current and needed breastfeeding knowledge among NICU healthcare providers in mainland China. The results indicate that providers have insufficient knowledge about breastfeeding preterm infants to support successful breastfeeding in mothers of premature infants. This finding is consistent with previous studies [6, 14–16] demonstrating that physicians and pediatric nurses lack the necessary breastfeeding knowledge and confidence to managing breastfeeding problems. Provider’s knowledge about breastfeeding on the 6 domains varied: they were relatively knowledgeable about indications and methods for breastfeeding preterm infants; however, they had an inadequate knowledge about the characteristics of premature human milk. Providers seemed more knowledgeable about the daily management and usefulness of breastfeeding for preterm infants but were less likely to know the specifics about the human milk composition and benefits for premature infants. Participants in this study were healthcare providers from tertiary care hospitals, which assumes they should have more clinical expertise and experience. However, results from this study indicate that many NICU providers have a knowledge deficit about breastfeeding preterm infants. Our findings reflected that the breastfeeding knowledge deficit of NICU health professionals in the smaller and/or rural hospitals may be more serious. Thus, it is urgently needed to increase provider’s knowledge and skills in breastfeeding preterm infants.

Many breastfeeding guidelines and systematic breastfeeding plans have been created for NICUs, with the goal of increasing the rates of breastfeeding preterm infants among developed or developing countries [17–21]. Currently, breastfeeding promotion programs have been introduced in NICUs across many districts in China. However, each program may be slightly different and not include all the relevant information necessary to increase a provider’s knowledge about breastfeeding. Some programs have training for healthcare professionals [22], but others do not [23]. This survey suggests that improving healthcare provider knowledge about breastfeeding preterm infants is an important aspect of providing support to mothers of preterm infants, which can improve rates of breastfeeding.

Findings in our study revealed that healthcare provider knowledge of breastfeeding among preterm infants was associated with age and total offspring, which is consistent with a previous study [6]. This finding suggests that with age comes experience, possibly work or personal experience with children or breastfeeding and this better equips providers to assist mothers of preterm infants with breastfeeding. Although work experience was not associated with breastfeeding knowledge, total offspring suggests that personal experience with breastfeeding increases knowledge. Furthermore, Karipis [16] noted mothers who had a successful breastfeeding experience showed higher level of breastfeeding knowledge than those without breastfeeding experience or failed breastfeeding experience.

NICU providers with more education and higher profession title scored higher on the knowledge questionnaire. To a certain extent, profession title may reflect the level of one’s professional knowledge and skills in China. Those with more knowledge or more clinical expertise, receive promotions. Along these lines, more education represents the extent of knowledge. Better educated individuals often value learning and continually seek out opportunities to gain more knowledge. Additionally, profession (physician or nurse) was associated with having a higher knowledge score. Physicians scored much higher than that of nurses. Again, the educational background of physicians is higher than nurses and they may have spent more time acquiring specific knowledge about preterm infants. Participants in this study consist mainly of nurses and providers with junior professional title, which may explain the overall low level of knowledge of providers.

Results of our study also showed that the majority of NICU staff felt lack of knowledge of breastfeeding and eager to get training on that. Indications and contraindications to breastfeeding premature infants was the most mentioned knowledge they want to learn. Expert lectures was considered as the most effective way to get these breastfeeding knowledge. Due to the time-saving and efficient nature to knowledge, ward rounds has been a major way for learning for healthcare providers. It is therefore recommended that thematic lectures including cutting-edge and targeted contents should be provided to NICU staff to raise their level of knowledge about breastfeeding preterm infants.

### Limitations

This study provides a baseline understanding about breastfeeding knowledge and training needs among NICU healthcare providers in China. Potential limitations of this study include the convenience sample of only 9 tertiary level NICUs from 4 districts in China. The sample is mainly comprised of nurses and providers with junior professional title and cannot be generalized to the healthcare providers with different demographic characteristics. In addition, the online nature of the survey cannot guarantee health professionals’ attention while completing the survey or limiting access to reference materials. However, based on the overall low scores from providers, the authors are somewhat confident that participants did not use additional materials to assist them in answering questions.

## Conclusions

Findings from this study suggest that NICU healthcare providers in China lack of sufficient knowledge about breastfeeding preterm infants and have a strong desire for more training about breastfeeding in this population. Further research is needed to confirm and expand these findings with a larger sample size throughout all hospitals in China. Studies could explore specific training programs and breastfeeding content that helps healthcare providers develop new knowledge about breastfeeding in the preterm infant population.

## Abbreviations

ANOVA: Analysis of Variance
CVI: Content validity index
NICU: Neonatal Intensive Care Unit
SD: Standard deviation
SE: Standard error
WHO: World Health Organization
